# Supplementary material for: Visuo-haptic processing of unfamiliar shapes: Comparing children and adults
Source: PLoS One. 2023 Oct 27;18(10):e0286905. doi: 10.1371/journal.pone.0286905 (PMC10610448; doi:10.1371/journal.pone.0286905)
Supplement: Attachment — Submitted filename: Response To Reviewers.docx [file pone.0286905.s001.docx]

In the following responses, each reviewer’s question is prefixed by R[reviewer number]Q[question number], and our answer as R[reviewer number]A[question number]

Reviewer 1

R1Q1: The article is original and well-written. The methodology is well done and the statistical analyses that have been performed are also adequate. the authors have also brought the most critical points into the discussion.
The only weak point of the article in my opinion is the small sample of adult subjects compared to children. Is it not possible to increase it? The results would be even more interesting

R1A1: Thank you for your comment and the positive feedback. As to the number of participants, we have added the following to the “Participants” section of the manuscript to clarify this:

“The sample size for the adult group was set similar to that of studies using this well-documented paradigms with adult participants (17-21) - the sample size for the children group was set to be larger given the potentially larger variability in performance and different attention span following previously published research (e.g., (10,12)).”

Minor revision:

R1Q2: page 10: The sentence "correlation results....." is not complete

R1A2: The sentence in the manuscript reads “Correlation results only pay attention to the relative differences in the rating profiles, but do not assess absolute differences in the rating behavior.” and seems complete? Perhaps the reviewer can clarify in which way the sentence was incomplete?

Reviewer 2

R2Q1: No justification was provided for the sample sizes (60 per group for children and 15 per group for adults)

R2A1: We have added the following to the “Participants” section of the manuscript to clarify this:

“The sample size for the adult group was set similar to that of studies using this well-documented paradigms with adult participants (17-21) - the sample size for the children group was set to be larger given the potentially larger variability in performance and different attention span following previously published research (e.g., (10,12)).”

R2Q2: There is no description of how the data was treated: each child participant provided 45 data points (45 comparisons) and each adult participant provided 90 data points (45 comparisons, twice). The authors only mention that ‘their responses were correlated to each other’ – were each individual ratings for each participant correlated to each individual rating for other participants? Was averaging performed across pairs that were more/less similar? Across each individual’s responses? Also, what type of correlation was calculated?

We apologize for not spelling this out completely and have added an Analysis part that aggregates the information of the result section to make this more transparent.

“Analysis:

The individual ratings of each participant for 45 trials (all trials for the children and only the first repetition for the adults) were analyzed in the following. Ratings were first correlated across all participants via Spearman correlations for all pair-wise comparisons within each modality group to check for inter-rater consistency. We next also correlated the responses for the 45 comparisons across participant groups (but within modality) for all pairwise comparisons to gauge how well response patterns would match. Bootstrapping was done to determine confidence intervals for these comparisons. Since correlations only look at relative changes in responses, we next conducted standard t-tests comparing the adults and children for each of the cells of the 9x9 (symmetric) similarity matrix for each condition, followed by correction for multiple comparisons.

An analysis of the same-object comparisons was conducted next to determine a ground-truth “accuracy” response as the similarity rating for these trials should be “5”. Two-sample t-tests were run to compare average same-trial ratings across modalities and groups. Linear regressions were then conducted for the children participants to see whether there would be any trend in accuracy as a function of age.

The next set of analyses focused on the similarity matrices and the resulting perceptual space. First, the similarity matrices were compared to those obtained from the actual physical parameters used during stimulus creation to check how well they would match. Next, multidimensional scaling (MDS, e.g., Cooke et al., 2009) was applied to investigate the topologies of the resultant perceptual spaces. Stress values of the MDS and bootstrapped fits of the spaces against each other using Procrustes analysis were performed to determine the degree of topological similarity.”

R2Q3: It is not clear if only first trial or both trials are included in the analysis of adults’ performance. If both trials are included: it is possible that this stabilized participants’ responses and led to the finding that responses were more reliable in adults. If both adult responses were included, it would be important to report the analysis of only the first response, as it may change the results and the authors’ interpretation / conclusion.

R2A3: We fully agree that only the first repetition (trial) should be analyzed and did so in the paper. As now stated more clearly in the analysis part (information that was previously in the Results and Discussion parts), only the first trial was analyzed and reported in the Results section. The second repetition is only used as a measure of consistency to explore the possibility of exposure effects, as mentioned further in the discussion (now with a short quantitative statement).

R2Q4: On page 8 the authors report that age differences were ‘not visible in the data’ – what statistical test was performed to support the statement?

R2A4: Because the results were presented in a correlation matrix, and sorted from youngest to oldest of the children tested, an improvement would have been qualitatively visible as a pattern in the correlation matrix colours (less correlated to more correlated). We have added the following to Page 9:

“In Figure 3, all results are sorted from the youngest to the oldest age to potentially highlight differences - any consistent change across age (such as improvement in consistency for older children) would be visible as a pattern in the correlation matrix colors - this, however, was not observed.”

R2Q5: On most figures the axes are not labeled.

R2A5: Most axes were either similarity matrix item numbers or arbitrary units for which labeling is typically not done. We did, however, omit to include labels for Figure 8, which is now redone with labels - we apologize for the oversight.

R2Q6: On page 4, figures 3 and 4 occlude text. This also occurs on page 10: Figure 6 occludes text and overlaps with Figure 5, occluding it as well.

R2A6: This may be a problem with the submission system - we have uploaded each figure separately from the manuscript as per the requirements of PLoS One. The captions were also resized to hopefully alleviate the problem.

R2Q7: On page 11-12, the authors perform two separate analyses on the averaged ratings for ‘same’ trials (visual vs. haptic in each age group, and then children vs. adults for each modality). The authors should select one set of comparisons.

On page 12 it is not clear what data is being used to ‘fit the response of all participants against age in months’.

R2A7: Two analyses were done to first check for differences in same-trial accuracy across adult and children participants and to, second, trace how or whether these responses would change with age for the children participants. Again, this is now more succinctly stated in the “Analysis” section.

R2Q8: Through the results section, the authors seem to begin interpreting their data (for example p 13 for age-related changes, p15 for estimated distances)

R2A8: We have added only intermediate conclusions as we think it would help to guide the reader through the various types of analyses.

R2Q9: On p. 14 it’s not clear whose data (adults, children, all) is being presented in the analysis of perceptual space. The same problem arises in the first paragraph for multidimensional scaling (page 16).

R2A9: We apologize for the oversight with the MDS stress values - the first paragraph relates to data from the children, whereas the later paragraph on Page 15 now also lists corresponding stress values for the adult data. The remaining paragraphs and figures are, however, labeled and introduced as to which participant group they refer to - perhaps the reviewer could point out which analysis was affected?

R2Q10: It’s often not clear why specific analyses are being performed, for example, on p 17 the authors say “To compare MDS results further across modalities…” They then indicate they left out participants for the calculation, but did not explain which participants or how they were selected for exclusion.

R2A10: We are sorry for the ambiguous wording - this is purely part of the standard bootstrapping calculation, in which variability of the measure is estimated by repeating the procedure with “left-out” data. This is now reworded as follows:

“This was done by repeating the analysis 1000 times, each time leaving out half of the participants for the calculation of MDS spaces and aggregating the results over repetitions.”

R2Q11: There is no report of how common specific children’s responses were about how they rated objects – though these are addressed in the discussion. It would therefore be important to describe these in the results

R2A11: Given the time-demands placed on us by the school, we were not able to collect these responses in a more structured fashion and hence just noted these down when children made comments about the objects after the experiment had already ended. We have added a comment to this effect to the manuscript on Page 7.

R2Q12: (data availability) The authors report that ‘some restrictions will apply’ but do not describe these restrictions. The authors also indicate that data will be made available upon publication via a Google share link.

R2Q12: Following PLoS One guidelines, data will be made available via an OSF link upon publication - this is what this refers to.

R2Q13: Generally the quality of the English language is appropriate. However, the constant use of numbers in the middle of sentences to refer to specific authors makes reading the manuscript awkward.

R2A13: We agree on the numbering issue - this will be changed during the editing phase, but does (unfortunately) adhere to the PLoS One citation style.

R2Q14: On page 4, the authors should add ‘participants’ after the second 15, and ‘the visual’ should be replaced by ‘the visual task’ in the sentence “A total of 15 participants performed the haptic task and 15 (participants) performed the visual (task).”

R2A14: Thank you - we have made the requested changes.

R2Q15: Further to the above, the authors should address the following:
Introduction: The introduction is very short (2 pages / 3 paragraphs), quite superficial, and lacks focus. The first paragraph briefly discusses unisensory development, and then multisensory development (which seems to be beyond the scope of this paper). The second paragraph first discusses older adults (which is again beyond the scope of this paper), how training can prevent decline (which seems again beyond the scope of this paper) and finally multisensory learning in children. Also, throughout the introduction (and the discussion), the authors use numbers to refer to their sources in the middle of sentences, essentially replacing the subject of verbs with numbers - for example “Similarly, in the visual domain (4,5) suggested that infants as young as 6 months old have the ability to retain visual stimuli…” This makes reading the paper quite awkward. The manuscript would benefit from a more clearly focused introduction.

R2A15: Please see above for the issue with numbering references. We had tried to focus the introduction (see also R2Q20 below), but there is - at present - not enough related work in the area of developmental research in this context, such that we opted to include the work on multisensory integration. We think, however, that the streamlined Analysis, Results, and Discussion section in our revision make for a better reading experience overall.

R2Q16: Discussion: The discussion is generally superficial and lacks clear explanations.
• The authors claim that “From the MDS results in Figure 11 it seems that the children may have relied more on fast-outstanding features than on less outstanding details.” What kinds of features and details are the authors referring to here (and how are features and details different)? How do the results support this interpretation? An explanation is needed. In the next sentence the authors refer the reader to the reconstruction in Figure 6 – but Figure 6 represents differences between adults and children’s ratings. They then refer back to the use of petal edges and stems and indicate these features were “recurrent criteria mentioned by children” – however the authors did not report any analysis of this information (they only mention them in the method) – a table of frequent comments would have been helpful, especially with a frequency count. The authors then say that “Changes in feature processing were also observed in (10).” It’s not clear what the authors mean or how those other findings relate to theirs.

R2A16: We apologize for being unclear about this. Our qualitative interpretation of these findings relate to the features that were mentioned by the children spontaneously during the experiment - unfortunately, we were not able to conduct structured post-experiment debriefings due to time constraints. This is now clarified on Page 7:

“Given the time constraints placed on this experiment, it was not possible to conduct a more detailed, structured post-experiment debriefing unfortunately.”

and in the discussion by adding that we conducted only a qualitative assessment and concluding as follows:

“A more in-depth analysis of the introspective features of children versus adults in this task, however, was not possible in the context of the present study, given the time constraints in running the experiment in the school and hence is left for future work.”

R2Q17: On page 20, the authors describe other research on verbal fluency and state that “This research could also explain why the children group in this experiment did not exhibit changes around a certain age…” How so? The authors should provide this explanation.

R2A17: We have added the following to the Discussion to clarify our thinking:

“ - this is because verbal fluency development would have an impact for a familiar object task, whereas the objects in our experiment were unfamiliar making it potentially more difficult to attach clear semantic associations.”

R2Q18: On page 21, the authors claim that “the second trial for adults did not show any kind of improvement in comparison to the first trial” – but no data or statistical analysis is reported

R2A18: This is now clarified and we have updated this to read as follows:

“One potential limitation of our experiment is that we were only able to gather results from a single trial per comparison due to time restrictions and concentration of the younger children. In this context, it is interesting to note that the second trial for adults did not show differences in comparison to the first trial (correlation of r=.87 of first repetition to second repetition in the ratings). Similarly, the children’s same trial ratings were consistent at similar levels to those of the adults, showing that the ratings per se were not unreliable.”

R2Q19: On page 21, the authors indicate that the children’s reliability may be an issue, but then say “we did observe a greater variability in ratings for children, and their ratings tended to be more similar in absolute terms.” This seems contradictory and needs to be clarified

R2A19: We apologize for this imprecisely stated sentence - the second part is not relevant and was now deleted. We did, however, observe greater variability in responses across children compared to adults (see Results section).

R2Q20: On page 21, the authors justify their choice of a similarity rating task – this justification would be more appropriate in the introduction.

R2A20: We have tried to fit this section into the introduction, but have reverted it back to the discussion in our revision. The introduction already contains a justification of why we chose to use this task based on prior work in adults on exactly this paradigm. The paragraph the reviewer refers to, however, relates more to potential issues with similarity ratings and their reliability (see previous answer), and we therefore felt that it would still be better suited for the discussion section.

R2Q21: The discussion also includes parts that seem irrelevant / beyond the scope of their paper. For example, on pages 21-22, they discuss hastening multisensory integration through training, which does not seem appropriate here.

R2A21: We agree and have now deleted this section.
