# Supplementary material for: Visuo-haptic processing of unfamiliar shapes: Comparing children and adults
Source: PLoS One. 2023 Oct 27;18(10):e0286905. doi: 10.1371/journal.pone.0286905 (PMC10610448; doi:10.1371/journal.pone.0286905)
Supplement: Attachment — Submitted filename: Response to Reviewers- Second Revision (1).docx [file pone.0286905.s002.docx]

In the following responses, each reviewer’s question is prefixed by R[reviewer number]Q[question number], and our answer as R[reviewer number]A[question number]

Reviewer 1

The article is very interesting especially because it concerns what happens in the developmental age. The authors have made good statistical analyses that are also well represented graphically.

However, I have three major concerns:

R1Q1: … there is a lack of description of the visual experiment (it is taken for granted that one understands what is being done but I cannot find any explanation and instead believe it would be useful to explain what is being done and asked of the subject)

R1A1: Since both experiments used a very similar setup, we had initially described them in an integrated fashion. We have now included two separate descriptions to facilitate understanding of the setup as follows into the Methods section:

Haptic Task: A fixture on a desk with a curtain was used during the haptic exploration through which the participants were asked to extend their dominant hand for exploration. During the main task, the objects were placed on the table at a comfortable handling distance of about 0.4m away (Fig. 2) where the participants explored them haptically for 6-8 seconds after which the first object was replaced by the second object for comparison. The rating was given verbally after two objects were presented and recorded by the experimenter.

Visual Task: During the visual experiment, the objects were placed in front of the participants at the same distance, and they were instructed to explore them visually only. Participants were allowed to move their head a little but otherwise were instructed to not get up from the chair during visual exploration. The exploration time for each object was kept similar to the haptic condition, and objects were kept behind a barrier to prevent participants from seeing them during the experiment. Ratings were again given verbally after each object pair and recorded by the experimenter.

R1Q2: I am very amazed that the haptic modality seems to be more helpful than the visual modality in children and so I wonder how they are made to see these shapes. the author's did not report any infomration about vision of the enrolled children. can you specifiy if they have all a normal vision, if the experimetn was done with glasses if they need ....

R1A2: Thank you for these comments. Concerning the first statement, we would like to add a clarification. We actually do not claim that the haptic modality is “more helpful” here. To summarize the main findings from the manuscript’s discussion briefly, we show:

- evidence for less response consistency in both visual and haptic groups in children compared to adults
- evidence for a small improvement of visual accuracy across the school age
- no evidence for larger-scale changes in response patterns across the school age otherwise
- distinct differences in the haptic perceptual space of children compared to that of adults, but much less so in the visual space

From this, we conclude in our discussion that there may still be development of haptic perceptual processing after the age range tested in our study.

Nonetheless, since conditions should be similar and comparable across visual and haptic groups, we also took great care to check that all children (and adults) had normal or corrected-to-normal vision (as well as no signs of neurological deficits). Thank you for pointing out that this information was not included in our manuscript - this is now added to the Participants section.

As an additional comment, the present data from the adults is very much in line with other (cited) studies that show similar processing of unfamiliar shapes in the haptic and visual modality (eg Cooke et al., 2007; Gaissert et al., 2010; Lee Masson et al., 2016) - this is mentioned in the introduction in the last paragraph.

R1Q3: I wonder if comparing a population of children from one country with that of adults from a country with very different habits can really be correct.

R1A3: Thank you for this comment. As stated in the previous answer, the ability of adults to reproduce the original space of the stimuli has been proven in previous experiments (across different cultures). At present we are not aware of any evidence showing strong cultural differences for Korean and German backgrounds in lower-level similarity processing. We kindly refer the reviewer to the studies of Cooke et al., 2007; Gaissert et al., 2010; Lee Masson et al., 2016.

R1Q4: In the introduction you refer to the age at which visual acuity and contrast sensitivity develops, but you don't specify whether you are talking about months or years. since in the previous paragraph the reference was to months I think it should be better specified what the authors mean here

R1A4: Thank you for this suggestion. Adjustments were made to the introduction to indicate years when talking about ages of children.
