# Supplementary material for: Visuo-haptic processing of unfamiliar shapes: Comparing children and adults
Source: PLoS One. 2023 Oct 27;18(10):e0286905. doi: 10.1371/journal.pone.0286905 (PMC10610448; doi:10.1371/journal.pone.0286905)
Supplement: Attachment — Submitted filename: PONE-D-22-15932_review.docx [file pone.0286905.s003.docx]

**REVIEW**

**Visuo-haptic processing of unfamiliar shapes: comparing children and adults**

This is an interesting paper on a timely topic, looking at the similarity in how adults and children rate objects they explore via touch versus via looking. Rather than consider integration between the senses, they consider a given each sensory modality individually. They focus on children 6-11 years of age as they highlight that changes in the processing of vision and touch have been established around 8 to 9 years of age. Importantly, they use ratings for unfamiliar objects where they parametrically vary stimulus dimensions for visual and 3D printed haptic objects, a total of 9 unique objects presented in all possible pairings, for 45 trials total. A 5 point Likert scale was used to obtain ratings.

While I thought the approach of the paper was strong, the methods and analyses were not always straight forward.

MAJOR COMMENTS

1.The authors had 30 adult participants, 15 in the haptic group and 15 in the vision group. Each adult completed 2 repetitions of the experiment and used a numerical scale to give their ratings of the similarity between 1 given pair of objects. Unlike the adult data, 126 children participated, with a final of 30 children in the haptic group and 30 children in the vision group. Each child ran only 1 repetition of the experiment and provided verbal ratings. How could these discrepancies contribute to the results of the analysis? How do verbal vs numerical ratings compare,? Why was this aspect of the study changed? Also, how did the authors determine that they needed to double the amount of data collected in children to account for the additional variance in younger cohorts?

The author’s state that the children’s age ranges from 6-11 (although figure 8 also seems to go to 125 months, or age 10.5 years), yet no range is provided for the adults, only the mean of 24.8 years is specified. It would be good to know the spread in age for both groups since it is stated that results are sorted by age. A broader spread in the age of child participants might contribute to more variability. Also, what was the exclusion criterion for not including data?

2. While IRB approval is mentioned for the adult study in Korea, no IRB approval is mentioned for studies run in Germany in the school setting. Was an IRB not obtained for this work?

3. It is great that the abstract shapes are created by parametrically varying certain stimulus dimensions. What those dimensions are is not clear in Figure 1. Some background as to how the parameterization was done and why should be provided.

4. The methods state that children were given 6-8 seconds to explore the first object before being given the second object and that adults were given 6 seconds. Was this sufficient time? The authors state that children found the task easy, but is there some measure of mean reaction time in their making their judgments? One would imagine children might need more time to explore the shapes, especially via touch, than adults.

5. It is not clear what the correlation matrices in Fig 3 and 4 are plotting. The x and y axes are not labelled. The text mentions that results are sorted by age, but it is not clear how this information is being represented. Also, Figure 3 and 4 should have a part (a) and (b), as mentioned in the text, but the figure is missing those labels.

6. What does the color coding in Figure 6 indicate? If it is level of significance, there should be clear indication of what each color means in terms of significance or size of the effect.

MINOR COMMENTS:

Highlighted in yellow are places where the authors use a number for a citation in an odd way or places where an article needs to be changed, with the changed word added.

Page 7: Change “to haptically fully explore (Haptic group)” 🡪 “to fully explore via touching (Haptic group) and then to fully explore via looking (Visual group)”

Page 15: The stress values obtained for judging the goodness-of-fit of a two-dimensional scaling solution were s = 0.04 for the haptic condition and s = 0.04 for the visual condition which is determined as a “good” fit. (17) (23). – extra period should be eliminated

In Figures, differences that are significant should have a clear label and the figure legend should indicate the p value. For example, Figure 13, it is not clear what the “+” indicates

Page 15: “The children’s performance was as good as the adults’ and there was no advantage OF age in the categorization task.

Page 19: “To summarize, in this experiment we looked into the developmental trajectory of haptic and visual processing FOR unfamiliar objects as opposed to the integration of both senses in elementary school children.
